# Paleodermatology: Dermatoscopic findings of “Niño del Plomo” an Incan mummy in Chile

**DOI:** 10.1016/j.jdcr.2023.10.009

**Published:** 2023-10-29

**Authors:** Verónica Catalán, Mario Castro, Raúl Cabrera, Verónica Silva-Pinto, Alex Castro, Cristóbal Lecaros

**Affiliations:** a Faculty of Medicine, Department of Dermatology, Clínica Alemana-Universidad del Desarrollo, Santiago, Chile; b Faculty of Medicine, Department of Dermatology, Universidad de Chile, Santiago, Chile; c National Museum of Natural History, Santiago, Chile; d Faculty of Medicine, Department of Morphology, Clínica Alemana-Universidad del Desarrollo, Santiago, Chile; e Doctoral Programme in Mediterranean Geography and History from Prehistory and Modern Age, Early Modern History Department, Faculty of History and Geography, Universitat de València, València, Spain; f Faculty of Medicine, Department of Pathology, Clínica Alemana-Universidad del Desarrollo, Santiago, Chile; g Dermatology Residency Program, Faculty of Medicine, Department of Dermatology, Clínica Alemana-Universidad del Desarrollo, Santiago, Chile

**Keywords:** acral external pigmentation, archeology, Beau’s lines, Chile, dermatoscopy, Inca civilization, lices, mummy, skin biology, subungual hematoma

## Introduction

Paleodermatology is a collaborative science that provides insights into the physiology and pathology of ancient populations as well as informs physico-chemical properties currently relevant to dermatologic research.^1^

Mummification is a taphonomic process antagonistic to putrefaction characterized by dehydration and desiccation of soft tissues. Spontaneous natural mummification requires arid conditions, such as extreme temperatures, dryness, and hypoxia, to prevent cell autolysis, insects from laying eggs, and bacteria growing on the cadaver. Once skin is mummified, it is extremely resistant to decomposition and can remain unchanged for thousands of years. Very well-preserved micro- and ultrastructures have been observed in mummified skin.^2^

## Case report

In 1954, near Santiago, Chile, at *El Plomo* mountain—located at an altitude of 5400 m above sea level (33°13′S - 70°13′W)—an ancient corpse of the pre-Hispanic American was discovered. Approximately 5 centuries following his death, a child was found frozen after being buried in the context of an Inca ritual human sacrifice, the *Capacocha*.^3^ According to archeological data, the body can be attributed to the Inca culture, and its approximate date is between AD 1480 and 1500.^4^ Nowadays, it is preserved at the Chilean National Museum of Natural History in controlled environmental conditions of temperature (–2 °C to –4 °C) and humidity <45%.

The authors had the opportunity to examine the mummy when transported under controlled conditions to a computed tomography for noninvasive body conservation assessment. The analysis was made using DermLite Foto X and DermLite DL4 devices (DermLite LLC). To assess the accuracy of dermatoscopic evaluation, a 5-mm punch skin sample was obtained from the plantar region of the left foot where an irregular black- to dark-brown patch was observed and exogenous pigmentation was suspected. The tissue was prepared following an established protocol^5^ and subsequently stained with hematoxylin-eosin and periodic acid–Schiff.

**Fig 1:**
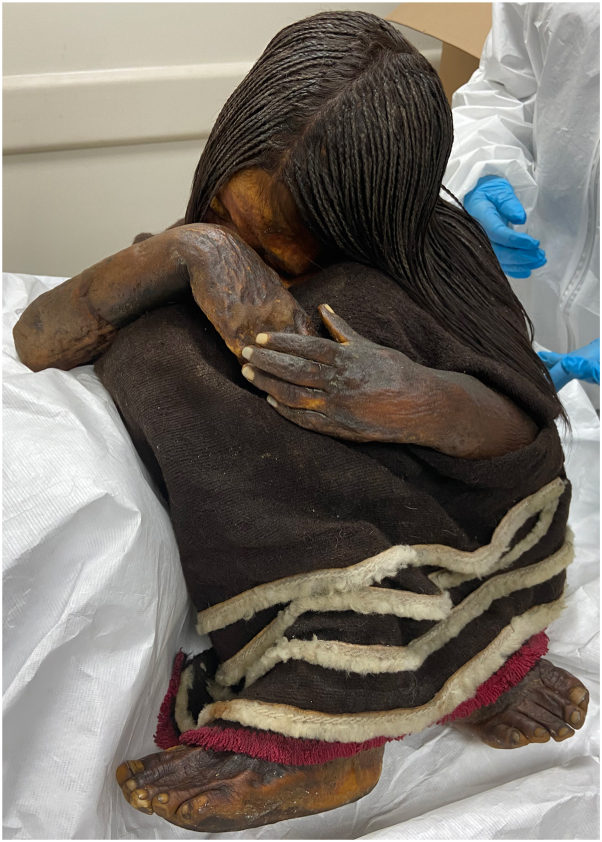
The mummy “El Niño del Plomo.” Spontaneous natural mummification of an 8-year-old infant.

The mummy was a healthy 8-year-old boy whose body was in a sitting position, hands wrapped around his knees with the head between his legs and the eyes closed (Fig 1). An exhaustive description of the physical anthropology of the mummy has been published previously.^4^ Sex determination was made by visualization of masculine genitalia, and age was estimated by analyzing the hand radiograph and according to the degree of development of the teeth.^3^

The mummified body preserved intact skin over its entire surface. On the arms and legs, the mummified skin presented a dark-brown hue and a rigid texture resembling lichenification. Hair and nails were preserved almost intact; however, some nails of the feet showed Beau’s lines. We observed scalp scales, empty and full nits from lice on the braided hair. The mummy showed hyperkeratosis and extensive hyperpigmentation of the plantar region of the feet. We noticed at the base of the thumb and index finger of the left hand a round pale erosion with the appearance of prior manipulation that corresponded to a previous skin sample of a human papillomavirus wart evaluated through electron microscopy.^3^ In the middle finger of the right hand, we observed bluish subungual blotches, consistent with subungual hematoma. These findings are presented in Supplementary Figure 1, *A–F* (available via Mendeley at https://data.mendeley.com/datasets/myn4bfgsdb/1).

**Fig 2:**
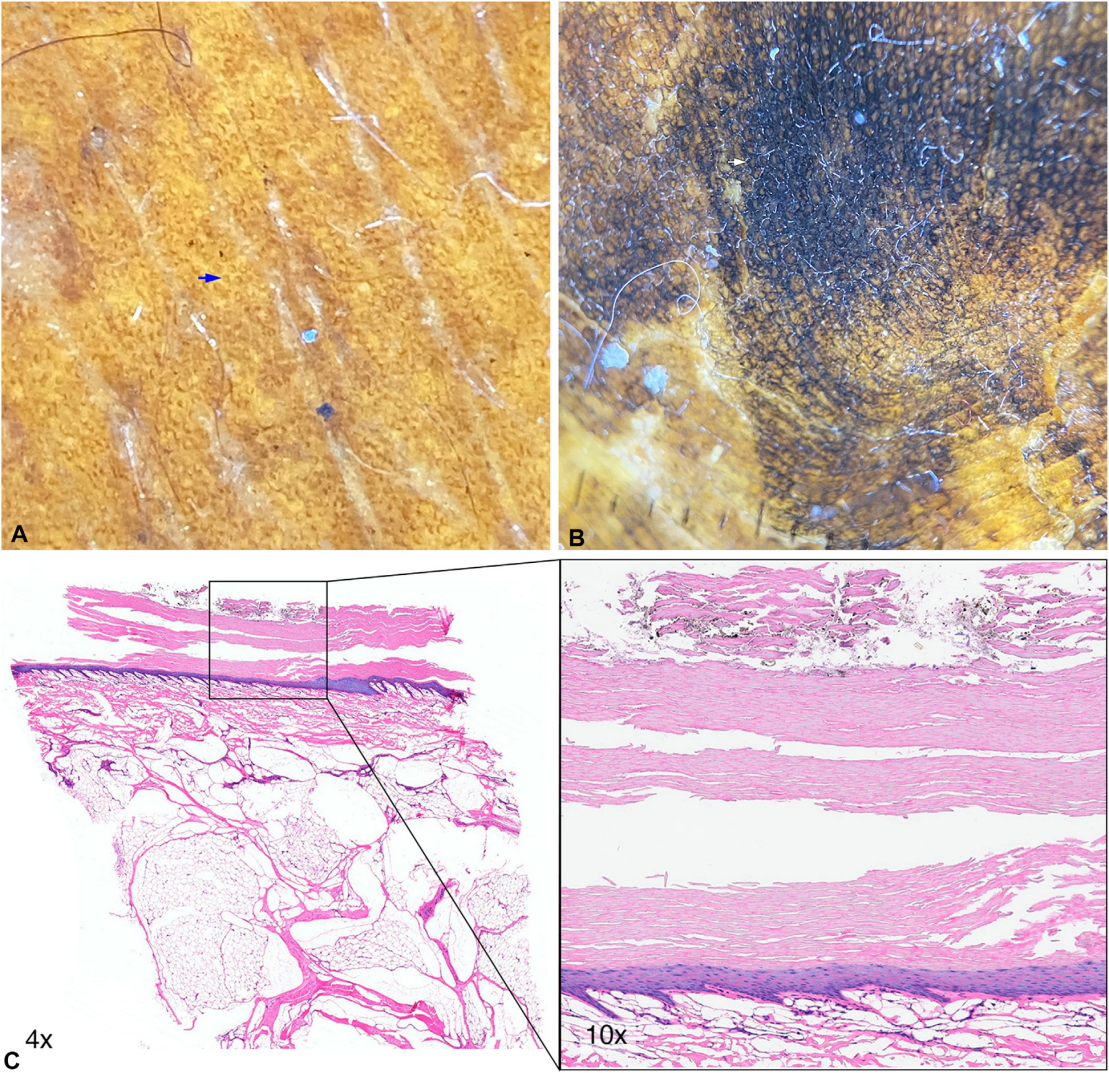
**A,** Dermatoscopy and histology of the mummy “El niño del Plomo.” Follicular openings and perifollicular pigmentation in skin from the trunk of the mummy (*blue arrow*). **B,** Exogenous pigmentation, dermatoglyphs, and acrosyringiums of the plantar region (*white arrow*). **C,** Histopathologic examination of plantar skin. The plantar skin sample showed thickening of the stratum corneum and black-brown particulate material of exogenous type in the superficial zone, consistent with dust particles. The melanin pigment status in the specimen was normal. (Hematoxylin-eosin stain; original magnifications: ×4 and ×10.)

Dermatoscopy of nonlesional skin from the arm of the mummy is in Fig 2, *A*. It shows follicular openings and perifollicular pigmentation that is characteristic of phototypes 3 to 4 (Eumelanin Human Skin Color Scale 75-100, intermediate high). The plantar region demonstrated an irregular black to dark-brown patch with a parallel ridge pattern, a lattice-like pattern, well-preserved dermatoglyphs and acrosyringiums without disruption within the epidermal ridges (Fig 2, *B*). Histopathologic examination of plantar skin demonstrated the preservation of the skin architecture with no evidence of degenerative or inflammatory processes. The plantar skin sample showed thickening of the stratum corneum and superficial exogenous black-brown particles (Fig 2, *C*). Under polarized light, there were some elements of refringent crystalloid appearance compatible with dust particles (Supplementary Fig 2, available via Mendeley at https://data.mendeley.com/datasets/myn4bfgsdb/1). Incidental fungal colonization was observed with no associated inflammatory signs.

## Discussion

Various dermatologic conditions have been reported in mummified skin remains. The most common findings are infections and infestations followed by tumors and less frequently inflammatory dermatoses. A survey of conditions on mummified skin is presented in Supplementary Table I (available via Mendeley at https://data.mendeley.com/datasets/myn4bfgsdb/1).

This mummy confirms the endemic presence of pediculosis and human papillomavirus. A previous stool analysis showed that this child also experienced trichuriasis,^4^ which could explain the Beau’s lines because of cyclic anemia as in other human remains.^1^

To be sacrificed, this boy walked approximately 2800 km from *El Cuzco* to *El Plomo*. These impressive geographic journeys were not exceptional, and there is genomic evidence that attests the migratory dynamics of the Inca culture throughout the southern cone.^6^ This explains the observed plantar hyperkeratosis being so young and the dark-brown exogenous pigmentation in the sole of his feet. Acral exogenous hyperpigmentation has been previously observed using dermatoscopy^7^ and it was confirmed by histology in our study.

Mummified skin tends to have a darker tone than live skin because of dehydration and consequent concentration of eumelanin along with the deposition of dust and minerals in the skin.^8^

Besides environmental factors that minimize physical decay, the preservation of the dermal architecture seen by dermatoscopy can be explained by the following 3 aspects of the skin barrier: (1) the geometric (tetrakaidecahedral) conformation of the epidermal keratinocytes, resulting in optimal natural space-filling shape with minimal surface area^9^; (2) the presence of desmosomes, tight junctions and an energetically stable arrangement of lipids in the extracellular space, allowing impermeability^10^; and (3) the structural integrity of dermal type I collagen, an extremely durable material that shows barely any changes in its molecular structure >5300 years after its generation.^2^ All this allows for an extremely resistant, impermeable, and elastic tissue when it is hydrated and equally resistant but rigid when dehydrated.

Our case demonstrates that the preservation of the skin architecture seen by dermatoscopy has the potential to allow the translation of *in vivo* dermatoscopic knowledge to mummified skin. Although the mummification process limits some aspects of clinical cutaneous evaluation, dermatoscopy of mummified skin provides a fundamental noninvasive evaluation tool for paleodermatology research.

## Conflicts of interest

None disclosed.
